# Role of increased neutrophil extracellular trap formation on acute kidney injury in COVID-19 patients

**DOI:** 10.3389/fimmu.2023.1122510

**Published:** 2023-03-27

**Authors:** In Soo Kim, Do Hyun Kim, Hoi Woul Lee, Sung Gyun Kim, Yong Kyun Kim, Jwa-Kyung Kim

**Affiliations:** ^1^ Department of Internal Medicine & Kidney Research Institute, Hallym University Sacred Heart Hospital, Anyang, Republic of Korea; ^2^ Division of Infectious Diseases, Department of Internal Medicine, Hallym University Sacred Heart Hospital, Anyang, Republic of Korea

**Keywords:** neutrophil extracellular traps, acute kidney injury, endothelial, COVID-19, inflammation, mortality

## Abstract

**Background:**

A strong association between elevated neutrophil extracellular trap (NET) levels and poor clinical outcomes in patients with coronavirus infection 2019 (COVID-19) has been reported. However, while acute kidney injury (AKI) is a common complication of COVID-19, the role of NETs in COVID-19-associated AKI is unclear. We investigated the association between elevated NETs and AKI and the prognostic role of NETs in COVID-19 patients.

**Methods:**

Two representative markers of NETs, circulating nucleosomes and myeloperoxidase-DNA, were measured in 115 hospitalized patients. Serum levels of interleukin [IL]-6, monocyte chemotactic protein-1 [MCP-1], plasma von Willebrand factor (vWF) and urinary biomarkers of renal tubular damage (β2-microglobulin [β2M] and kidney injury molecule 1 [KIM-1]) were measured.

**Results:**

AKI was found in 43 patients (37.4%), and pre-existing chronic kidney disease (CKD) was a strong risk factor for AKI. Higher circulating NET levels were a significant predictor of increased risk of initial ICU admission, in-hospital mortality (adjusted HR 3.21, 95% CI 1.08–9.19) and AKI (OR 3.67, 95% CI 1.30-10.41), independent of age, diabetes, pre-existing CKD and IL-6 levels. There were strong correlations between circulating nucleosome levels and urinary KIM-1/creatinine (r=0.368, p=0.001) and β2M (r=0.218, p=0.049) levels. NETs were also strongly closely associated with serum vWF (r = 0.356, p<0.001), but not with IL-6 or MCP-1 levels.

**Conclusions:**

Elevated NETs were closely associated with AKI, which was a strong predictor of mortality. The close association between NETs and vWF may suggest a role for NETs in COVID-19-associated vasculopathy leading to AKI.

## 1 Introduction

Neutrophil extracellular traps (NETs) are extracellular webs of DNA, histones, microbicidal proteins, and oxidant enzymes released by activated neutrophils in response to various stimuli, including respiratory viruses and inflammatory cytokines (1). While NETs are thought to have an antimicrobial function in innate immunity, their dysregulation can initiate and propagate inflammation and thrombosis, causing severe tissue injury (2). In patients with influenza A infection, high levels of NETs predict a poor prognosis, and the inhibition of neutrophils and NETs is protective in several models of influenza-associated acute respiratory distress syndrome (ARDS) (3, 4). The presence of NETs in patients with coronavirus infection 2019 (COVID-19), the disease caused by SARS-CoV-2 infection, was first reported in 2020 (5, 6), with subsequent studies showing an association between circulating markers of NETs and clinical outcome (6, 7). In COVID-19, NETs were shown to be a prognostic marker (8).

Acute kidney injury (AKI) is characterized by a rapid decline in the estimated glomerular filtration rate (eGFR), accompanied by reduced renal blood flow, endothelial dysfunction, and tubulointerstitial inflammation (9–12), which together result in a high risk of death in hospitalized patients (13, 14). In COVID-19 patients, the prevalence of AKI ranges from 10% to35%, but is as high as 50% in those with severe disease and those in the intensive care unit (ICU) (15–17). Furthermore, consistent with AKI from other causes, COVID-19 AKI is associated with adverse outcomes, as the risk of all-cause mortality is ≥ 5-fold higher in COVID-19 patients with AKI than in those without (8, 18, 19).

Neutrophil dysregulation and excessive NET formation have been implicated in the development of organ damage. For example, interstitial NETs and NET-associated intravascular thrombi are characteristic features of ARDS in the lungs of patients with lethal COVID-19 (3, 20, 21). Accordingly, a role for NETs in the pathogenesis of COVID-19 AKI is likely to lead to tubular injury, excessive inflammation, and intravascular immune thrombosis (22–26). However, how NETs might induce AKI in COVID-19 is unclear, as are the prognostic implications of NETs in COVID-19 AKI.

In a previous study, we showed that circulating NETs levels are significantly higher in dialysis patients than in the general population and are a strong prognostic marker for mortality (27). In this study, we investigated the association between higher NET levels and AKI in hospitalized patients with COVID-19. We also investigated the relationship between NETs and various inflammatory parameters and the prognostic role of NET-related COVID-19 AKI on in-hospital mortality.

## 2 Methods

### 2.1 Study population and blood sampling

The study population consisted of 115 patients with COVID-19 diagnosed between January 2022 and May 2022. Diagnosis was based on reverse transcriptase-polymerase chain reaction detection of viral RNA from nasopharyngeal swabs from patients with clinical symptoms. Patients aged <18 years of age were excluded. Blood and urine samples were collected within 48 h of admission using EDTA tubes (367835, BD, Franklin Lakes, NJ, USA) for plasma and Serum Separator Clot Activator tubes (456073, BD) for serum. Plasma was obtained by centrifugation of blood samples at 1500 g for 10 minutes at 4°C, and serum was obtained by incubation at room temperature for 20-30 minutes followed by centrifugation as described for plasma. Both plasma and serum were aliquoted and stored at -70°C until analysis. This study was approved by our Institutional Ethics Committee (IRB No 2022-02-014). Informed consent was obtained from all study participants or, in the case of incapacity, from their next of kin.

Demographic information, data on comorbidities, and the history of vaccination against COVID-19 at the time of admission were extracted from the patients’ medical records. Initial vital signs, information on ICU admission, and the need for mechanical ventilation (MV) were also determined. The cycle threshold value was used to measure viral load. Biochemical parameters, white blood cell (WBC) count, neutrophil count, lymphocyte count, platelet count, albumin levels, the neutrophil/lymphocyte ratio (NLR), and platelet/WBC ratio (PWR), and serum procalcitonin, lactic acid, and brain natriuretic peptide (BNP) levels, were obtained from the patients’ medical records.

Inflammatory cytokines were further assessed by measuring serum levels of interleukin (IL)-6, high-sensitivity C-reactive protein (hs-CRP), and monocyte chemotactic protein-1 (MCP-1) levels, using the appropriate kits according to the manufacturer’s instructions. The levels of these cytokines were determined using ELISA kits (R&D Systems, Minneapolis, MN, USA).

### 2.2 Diagnosis of AKI

AKI at the time of admission was diagnosed according to the Kidney Disease: Improving Global Outcomes (KDIGO) consensus definition for AKI, which includes the serum creatinine (SCr) level and urine output. The use of Scr has been reported to be very accurate in predicting COVID-19 AKI (28). The presence of underlying chronic kidney disease (CKD) was determined based on laboratory data obtained in our hospital (n=81) within the previous 2 years. For the 34 patients without previous laboratory data in our hospital, the presence of CKD was determined from their medical history. In addition, SCr level was measured serially during hospitalization, and AKI was diagnosed retrospectively if the SCr level improved and the urine volume increased after intravenous hydration.

Urinary specimens at the time of admission were stored at -80’C until thawed for measurement of biomarkers of renal tubular cell damage. We used two markers, β2-microglobulin (β2M) and kidney injury molecule 1 (KIM-1), which are known to be associated with AKI.

### 2.3 Measurement of NETs and marker of endothelial damage

*In vivo* NET levels were quantified by measuring plasma levels of circulating nucleosome (histone-DNA) and MPO-DNA (Cell Death Detection ELISA Plus Kit; Roche Diagnostics, Basel, Switzerland) levels, as described in our previous paper (27). The degree of endothelial injury or damage was assessed by plasma von Willebrand factor (vWF) levels determined using a commercially available ELISA kit (Ray Biotech, Peachtree Corners, GA, USA).

### 2.4 Study endpoints

The primary outcome was in-hospital mortality according to baseline NET levels. The duration of hospital stays, ICU admission rates, and COVID-19 AKI occurrence were also compared between higher and lower NET groups.

### 2.5 Statistical analysis

Variables with normal distributions, confirmed in Kolmogorov-Smirnov tests, were expressed as the mean ± standard deviation (SD). Categorical variables were expressed as percentages and were compared in chi-squared tests. An independent sample t-test was used to identify differences among groups based on continuous values. Pearson’s correlation coefficient was calculated for circulating nucleosomes, MPO-DNA, IL-6, BNP, various biochemical factors, and comorbidities. Multiple logistic regression analyses were performed to evaluate the role of increased NET levels as a determinant of AKI. Cumulative survival curves were derived using the Kaplan-Meier method; differences between survival curves were compared using a log-rank test. A Cox proportional hazards model was used to identify independent factors in the development of the study’s endpoints. The predictive value was expressed as a hazard ratio (HR) with the corresponding 95% confidence intervals (CIs). A p-value <0.05 was defined as indicating significance. All statistical analyses were performed using SPSS version 24.0 (IBM Corp., Armonk, NY, USA).

## 3 Results

### 3.1 Baseline characteristics

The 115 patients included in the analysis had a mean age of 67.6 ± 17.1 years, with ~50% of the patients being older than 70 years. Of the COVID-19 patients, 28 (24.3%) died. Differences in baseline demographic and clinical characteristics according to mortality are described in **Table 1**. Patients who died during hospitalization were significantly older than survivors and had unstable vital signs on admission (low oxygen saturation, high heart rate, and high respiratory rate). As expected, the rate of ICU admission rate and the need for MV were much higher in these patients. Of all patients, 75 (65.2%) had received a COVID-19 vaccination prior to admission. However, COVID-19 vaccination was not associated with mortality, and the viral load measured at the time of admission did not differ between patients who died and those who survived. However, the time from the last vaccination to infection was significantly longer in the former (152 vs. 111 days, p=0.022). Hypertension (56.5%) and diabetes (40.9%) were the most common comorbidities, but neither was associated with mortality. Pre-existing CKD was present in 35 (30.4%) patients, with a significantly higher prevalence of CKD and pre-existing heart failure (HF) in patients who died than in those who survived (53.6% vs. 23.0% for CKD, and 57.1% vs. 14.9% for HF). Other comorbidities were similar between the two groups.

**Table 1:** Baseline characteristics according to mortality.

Variables	Total (n = 115)	Mortality		p
		Survivor (n = 87)	Death (n = 28)	
Age (years)	67.6 ± 17.1	66.4 ± 16.8	72.6 ± 17.2	0.046
> 70 years	57 (49.6)	38 (43.7)	19 (67.9)	0.022
Gender, Male, n (%)	63 (54.8)	49 (56.3)	14 (50)	0.356
BMI, kg/m^2^	23.4 ± 5.7	23.9 ± 5.9	21.9 ± 4.8	0.100
Vital Signs				
SBP, mmHg	131.1 ± 28.8	131.1 ± 2.4.4	130.9 ± 40.0	0.972
DBP, mmHg	76.7 ± 16.9	77.7 ± 14.1	73.6 ± 23.8	0.268
MBP, mmHg	95.5 ± 18.5	95.8 ± 15.3	94.4 ± 26.3	0.730
HR (/min)	92 ± 22	88 ± 20	102 ± 27	0.007
RR (/min)	22 ± 4	21 ± 4	23 ± 4	0.034
Saturation, room air (%)	91.6 ± 10.2	93.8 ± 7.7	84.7 ± 13.5	<0.001
ICU admission, n (%)	54 (47.0)	32 (36.8)	22 (78.6)	<0.001
Mechanical ventilation, n (%)	22 (19.1)	8 (9.2)	14 (50.0)	<0.001
Previous vaccination, n (%)				0.539
None	40 (34.8)	30 (34.5)	10 (35.8)	
1-2	32 (27.8)	23 (26.4)	9 (32.1)	
≥ 3	43 (37.4)	34 (39.5)	9 (32.1)	
Time from last vaccination, day*	120 ± 67	111 ± 57	152 ± 87	0.022
Viral load				
Ct_value_E_gene	23.0 ± 6.0	23.3 ± 6.4	23.6 ± 6.5	0.407
Ct_value_RdRp_gene	23.2 ± 6.1	22.2 ± 4.6	22.2 ± 4.5	0.309
Comorbidities				
Diabetes, n (%)	47 (40.9)	39 (44.8)	8 (28.6)	0.096
Hypertension, n (%)	65 (56.5)	48 (55.2)	17 (60.7)	0.386
Coronary artery disease, n (%)	16 (13.9)	10 (11.5)	6 (21.4)	0.156
Heart failure, n (%)	29 (25.2)	13 (14.9)	16 (57.1)	<0.001
Cerebrovascular disease, n (%)	19 (16.5)	15 (17.2)	4 (14.3)	0.485
Chronic kidney disease, n (%)	35 (30.4)	20 (23.0)	15 (53.6)	0.003
COPD, n (%)	5 (4.3)	3 (3.4)	2 (7.1)	0.353
Liver cirrhosis, n (%)	5 (4.3)	3 (3.4)	2 (7.1)	0.353
Malignancy, n (%)	25 (21.7)	17 (19.5)	8 (28.6)	0183
Dementia, n (%)	19 (16.6)	15 (17.2)	4 (14.3)	0.639
Long-term care facilities, n (%)	13 (11.3)	8 (9.2)	5 (17.8)	0.297
AKI at admission, n (%)	43 (37.4)	27 (31.0)	16 (57.1)	0.009
Total hospital stays, days	16.1 ± 11.4	14.2 ± 12.5	22.0 ± 18.1	0.017

*among 75 patients with vaccination, BMI, body mass index; SBP, systolic BP; DBP, diastolic BP; HR, heart rates; RR, respiratory rates; ICU, intensive care units; Ct-value, the cycle threshold value; COPD, chronic obstructive lung disease; AKI, acute kidney injury.

Baseline biochemical parameters according to mortality are compared in **Table 2**. In patients who died during hospitalization, the WBC count, neutrophil count, and N/L ratio were significantly higher but the PWR and albumin levels were lower than in surviving patients. In addition, the levels of lactic acid, inflammatory cytokines, IL-6, hs-CRP, MCP-1, and procalcitonin were significantly higher in the patients who died than in those who survived.

**Table 2:** Biochemical parameters by median NET level and mortality.

Variables	Mortality		p	Nucleosome, median		p
	Survivor (n = 87)	Death (n = 28)		Low (n = 56)	High (n = 59)	
NET markers*						
Nucleosome, OD	0.90 (0.47-1.68)	1.78 (1.19-2.0)	0.010	0.53(0.37-0.82)	1.89 (1.5-2.2)	<0.001
MPO-DNA, OD	0.21(0.13-0.38)	0.33 (0.12-0.5)	0.034	0.18(0.13-0.35)	0.33 (0.1-0.6)	0.003
Biochemical parameters						
WBC,/ul	8397 ± 4642	11216 ± 5475	0.008	7592 ± 3742	10576 ± 5536	0.001
Hemoglobin, g/dL	11.8 ± 2.4	10.9 ± 1.9	0.081	12.0 ± 2.5	11.3 ± 2.2	0.134
Neutrophil,/ul	6470 ± 4515	9418 ± 5595	0.006	5713 ± 3584	8657 ± 4588	0.001
Lymphocyte,/ul	1201 ± 752	940 ± 687	0.112	1148 ± 743	1137 ± 752	0.888
Platelet, x10^3^/ul	219 ± 97	164 ± 114	0.016	220 ± 101	184 ± 105	0.079
N/L ratio	7.1 ± 5.4	17.4 ± 10.6	<0.001	6.8 ± 5.3	12.3 ± 13.0	0.012
P/W ratio	30.7 ± 14.8	17.2 ± 8.9	<0.001	32.5 ± 16.8	22.6 ± 10.9	<0.001
Albumin, g/dL	3.8 ± 0.5	3.3 ± 0.7	0.003	3.8 ± 0.6	3.4 ± 0.5	0.035
Lactic acid	1.8 ± 1.3	3.0 ± 2.6	0.007	1.8 ± 1.1	2.4 ± 1.8	0.149
BNP, pg/mL	169 ± 330	1202 ± 1523	<0.001	256 ± 678	601 ± 1022	0.032
BUN, mg/dL	23.2 ± 16.2	34.8 ± 22.4	0.004	21.4 ± 17.1	29.7 ± 15.6	0.017
Creatinine, mg/dL	1.7 ± 1.8	2.7 ± 2.0	0.013	1.4 ± 1.0	2.4 ± 2.1	0.025
eGFR, at admission	64.5 ± 31.3	41.9 ± 31.3	0.001	66.8 ± 55.5	52.5 ± 32.8	0.018
<60 mL/min/1.73m^2^	35 (40.2)	19 (67.89)	0.010	22 (39.3)	32 (54.2)	0.078
<45 mL/min/1.73m^2^	26 (29.9)	17 (60.7)	0.004	16 (28.6)	27 (45.8)	0.040
<30 mL/min/1.73m^2^	16 (18.4)	16 (57.1)	<0.001	10 (17.9)	22 (37.3)	0.017
Need for RRT**	10 (11.5)	8 (28.6)	0.036	5 (8.9)	13 (22.0)	0.046
Inflammatory markers						
IL-6, pg/mL***	3.2 ± 1.4	4.6 ± 1.2	<0.001	3.4 ± 1.4	3.7 ± 1.5	0.251
MCP-1, pg/ml	88.3 ± 61.5	128.4 ± 77.9	0.008	89.1 ± 61.4	105.9 ± 72.5	0.204
Procalcitonin, ng/mL***	-1.7 ± 1.9	-0.1 ± 2.0	<0.001	-1.7 ± 1.9	-0.9 ± 2.1	0.027
Endothelial damage marker						
vWF, (μg/mL)	14.2 ± 9.5	15.6 ± 9.2	0.441	11.5 ± 7.8	17.2 ± 9.8	0.002

All data are expressed as mean ± SD except for those with *, which are expressed as median with range. **including both AKI patients with dialysis and CKD patients undergoing chronic dialysis ***log-transformed.

MPO, myeloperoxidase; WBC, white blood cell; N/L, neutrophil/lymphocyte; P/W, platelet/WBC; BNP, B-type natriuretic peptide; BUN, blood urea nitrogen; IL-6. Interleukin-6; MCP-1, monocyte- chemoattractant protein-1; vWF, von-Willebrand Factor.

### 3.2 AKI in COVID-19 infection

On admission, 43 patients (37.4%) had AKI, and the prevalence of AKI was significantly higher in patients who died than in those who survived (57.1% vs. 31.0%, p=0.009) (**Table 1**). Approximately one third of deceased patients required renal replacement therapy. AKI was also common in patients with pre-existing CKD or HF (60.0% in CKD vs. 26.3% in non-CKD, p=0.001, 40.5% with HF vs. 16.4% without HF, p=0.005).

**Figure 1:**
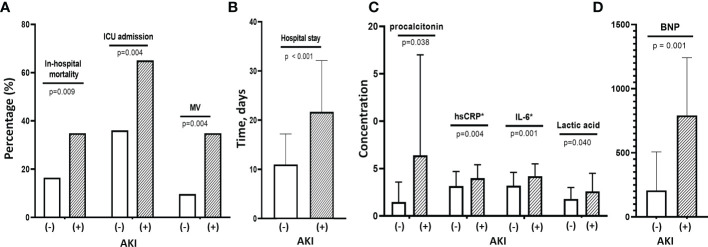
Clinical findings and outcomes associated with COVID-19 AKI. **(A)** Patients with COVID-19 AKI had significantly higher rates of in-hospital mortality, initial ICU admission and use of MV compared with patients without AKI. **(B)** These patients with AKI also had longer hospital stays than those without AKI. **(C)** Baseline levels of inflammatory cytokines such as hsCRP, IL-6 and procalcitonin as well as lactic acid levels were significantly higher in patients with AKI than in those without. **(D)** They also had higher baseline BNP levels than those without AKI. MV, mechanical ventilation; AKI, acute kidney injury; hsCRP, high-sensitivity C-reactive protein; IL-6, interleukin-6; BNP, B-type natriuretic peptide.

COVID-19 AKI was very strongly associated with poor outcome (**Figure 1**). Patients with AKI had significantly higher rates of in-hospital mortality (38.1% vs. 16.4%, p=0.009), initial ICU admission (64.3% vs. 37.0%, p=0.004) and MV (33.3% vs. 11.0%, p=0.004) and a significantly longer hospital stay (22.4 ± 19.5 vs. 12.4 ± 10.0 days, p<0.001) than those without AKI (**Figures 1A, B**). A comparison of COVID-19 AKI and non-AKI patients at baseline showed that WBC (11,000 ± 5,760 vs. 7,900 ± 4,120) and neutrophil (9,280 ± 5,777 vs. 5,980 ± 3,940) counts were significantly higher (p=0.001 and p<0.001) and PWR was significantly lower (20.7 ± 9.0 vs. 31.2 ± 16.2, p<0.001) in the former. In addition, the levels of all inflammatory markers, including serum ln_IL-6 (4.2 ± 1.4 vs. 3.2 ± 1.4, p=0.001), ln_hs-CRP (4.0 ± 1.4 vs. 3.1 ± 1.5, p=0.004), blood lactate (2.6 ± 1.6 vs. 1. 8 ± 1.2, p=0.040), procalcitonin (6.4 ± 11.4 vs. 1.1 ± 2.4, p=0.038) and BNP (792.5 ± 601.4 vs. 207.5 ± 311.4, p=0.001) were significantly higher in patients with AKI than in those without AKI (**Figures 1C, D**). For urinary biomarkers, urinary KIM-1/creatine (p=0.022) and β2M (p=0.006) levels were significantly higher in patients with AKI compared to non-AKI patients.

### 3.3 NETs, AKI and mortality in COVID-19 infection

**Figure 2:**
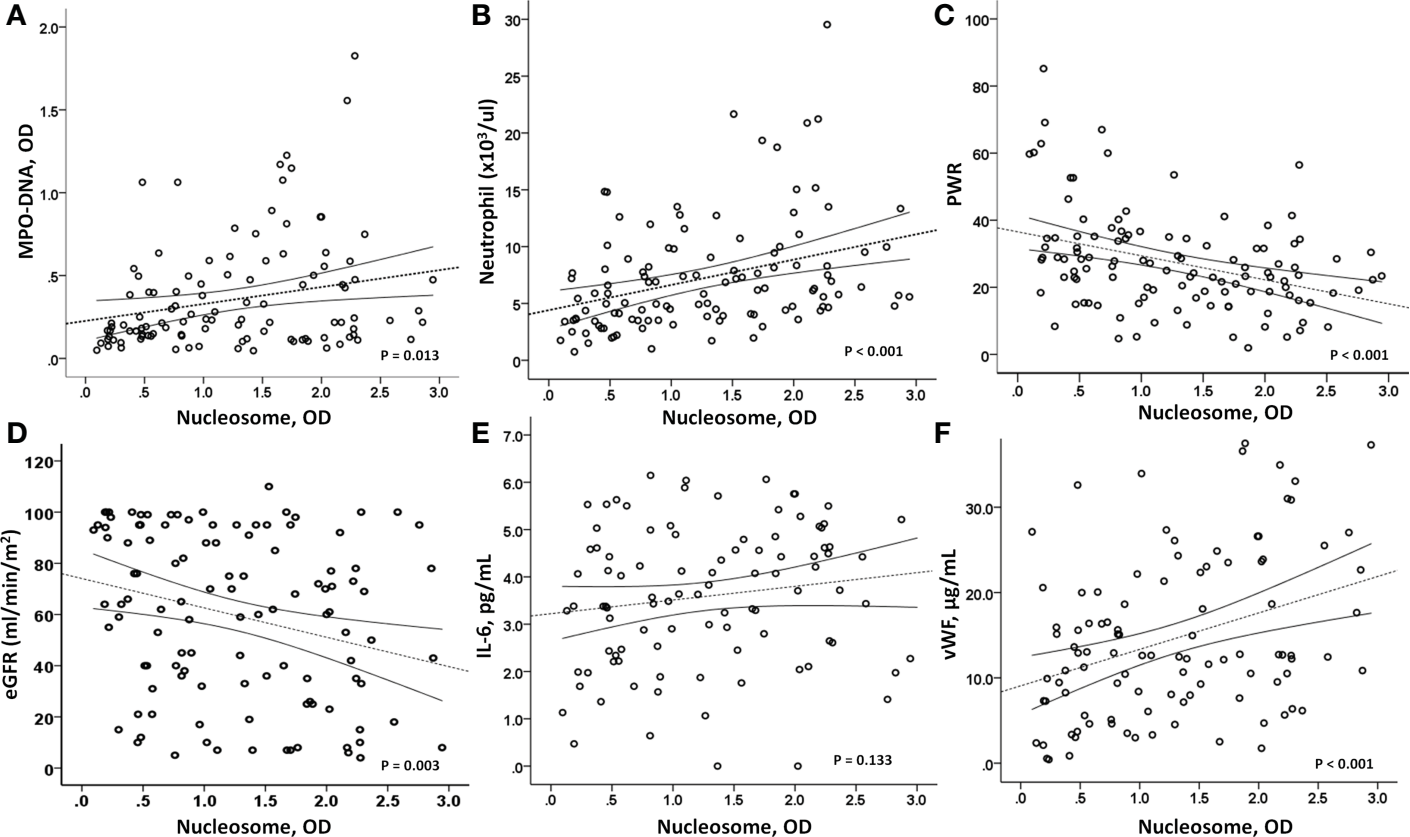
Correlation analysis. **(A)** As a marker of NETosis, circulating nucleosome levels were closely associated with MPO-DNA levels (p=0.013). **(B, C)** In addition, a strong correlation between circulating nucleosome levels and peripheral neutrophils (p<0.001) and platelet/WBC ratio (p<0.001) was observed. **(D)** And the nucleosome levels were inversely associated with renal function, eGFR (p=0.003). Interestingly, **(E)** nucleosome levels did not correlate with IL-6 (p=0.133), but **(F)** a strong positive association was observed with vWF levels (p<0.001), suggesting a close relationship between high nucleosome levels and endothelial dysfunction. MPO-DNA, myeloperoxidase-DNA; PWR, platelet/WBC ratio; eGFR, estimated glomerular filtration rate; IL-6, interleukin-6; vWF, von-Willebrand factor.

In the COVID-19 patients, we measured two representative markers of NETs, circulating nucleosomes and MPO-DNA, and both were closely associated (**Figure 2A**). Parameters associated with circulating nucleosome levels were previous coronary artery disease (r=0.185, p=0.048), HF (r=0.241, p=0.010), WBC count (r=0.258, p<0.001), neutrophil count (r=0.348, p<0.001), PWR (r=−0.347, p<0.001), and eGFR (r=-0.272, p=0.003), (**Figures 2B–D**). In contrast, the levels of inflammatory markers, including serum IL-6, hs-CRP, and MCP-1, did not correlate with circulating nucleosome levels (**Figure 2E**). Only procalcitonin levels showed a marginally significant correlation (r=0.173, p=0.072). However, in contrast to inflammatory cytokine levels, higher nucleosome levels had a strong positive correlation with vWF levels (r=0.356, p<0.001), a marker of endothelial damage (**Figure 2F**). This finding suggests an association between NETs and endothelial damage. There were no significant correlations between vWF and either the IL-6 or MCP-1 levels.

**Figure 3:**
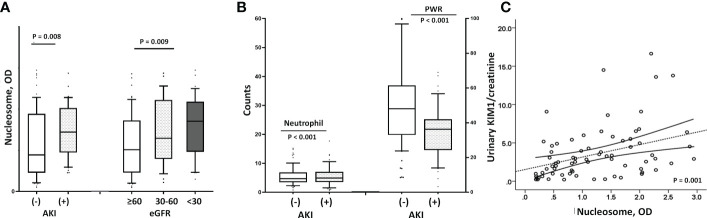
Relationship between renal dysfunction and nucleosome levels. **(A)** Patients with AKI had higher nucleosome levels than those without [median with IQR; 1.57 (1.05-1.81) vs. 0.87 (0.45-1.79), p=0.008], and the lower the eGFR, the higher the nucleosome level (p=0.009). **(B)** Similar to the findings with circulating nucleosomes, patients with COVID-19 AKI had significantly higher peripheral neutrophil counts (9288 ± 5777 vs. 5979 ± 3948, p<0.001) and lower PWRs (20.8 ± 9.0 vs. 31.3 ± 16.2, p<0.001) than those without AKI. **(C)** Urinary marker of AKI, the urinary KIM1/creatinine ratio also correlated significantly with higher nucleosome levels (r=0.368, p=0.001). AKI, acute kidney injury; eGFR, estimated glomerular filtration rate; KIM1/creatinine, kidney injury molecule 1/creatinine; BNP, B-type natriuretic peptide.

In addition, higher levels of NETs were significantly associated with AKI. As shown in **Figure 3A**, patients with COVID-19 AKI had significantly higher circulating nucleosomes (p=0.008) and peripheral neutrophil counts (p<0.001) but lower PWR (p<0.001) compared to patients without AKI (**Figure 3B**). The prevalence of AKI was 50.8% in the high NET group but 21.4% in the low NET group (p=0.001). Supporting these findings, urinary markers of AKI, urinary KIM-1/creatine (r=0.368, p=0.001) and β2M (r=0.218, p=0.049) levels were well-correlated with circulating nucleosome levels (**Figure 3C**).

**Figure 4:**
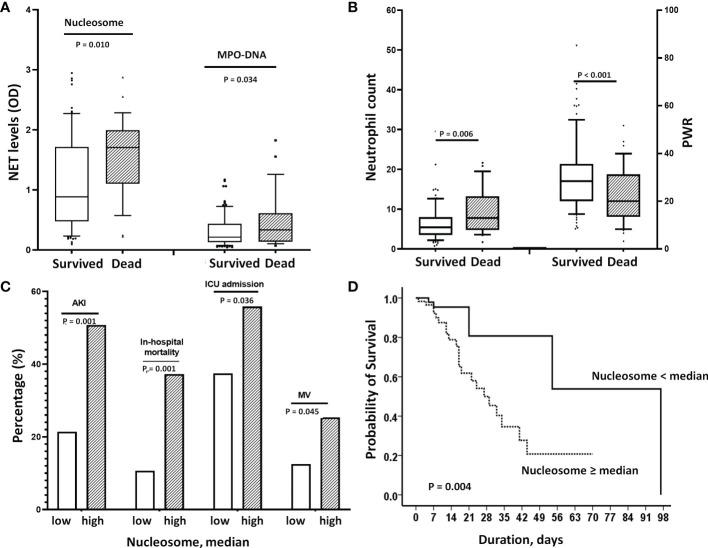
Effect of elevated NETs on mortality. **(A)** Nucleosome and MPO-DNA levels were significantly higher in patients who died than in those who survived [nucleosome: 1.78 (1.19-2.0 vs, 0.90 (0.47-1.68), p=0.010, MPO-DNA: 0.33 (0.12-0.5) vs. 0.21(0.13-0.38), p=0.034]. **(B)** Similarly, deceased patients had higher neutrophil counts (p=0.006) and lower PWRs (p<0.001) than survivors. **(C)** When nucleosome levels were divided into 2 groups according to median levels, patients in the high nucleosome group had worse clinical outcomes; significantly higher rates of AKI, in-hospital mortality, initial ICU admission and use of MV. **(D)** Kaplan-Meier analysis showed that higher nucleosome levels (>median) were associated with increased in-hospital mortality. In the adjusted Cox’s proportional hazards regression model, it significantly increased the risk by 3.2-fold. NET, neutrophil extracellular trap; PWR, platelet/WBC ratio; AKI, acute kidney injury; ICU, intensive care unit; BNP, MV, mechanical ventilation.

We also found that the NET markers, nucleosomes and MPO-DNA, were significantly higher in patients who died than in those who survived (**Figure 4A**). Similarly, there were significant differences in peripheral neutrophil counts and PWR between the two groups (**Figure 4B**). When the nucleosome levels were divided into two groups based on the median levels, patients in the higher NET group had significantly worse clinical outcomes than those in the lower NET group (**Figures 4C, D**). In a Cox regression analysis, higher NET level was independently associated with the risk of in-hospital mortality (unadjusted HR 3.81; 95% CI 1.43–10.16, p=0.007). Even after adjustment for risk factors, higher NET level remained a significant predictor of death (HR 3.21, 95% CI 1.08–9.19, p=0.035) (**Table 3**).

**Table 3:** Clinical Factors Influencing In-Hospital Mortality in COVID-19 Patients.

	Unadjusted		Adjusted	
Mortality	HR (95% CI)	P	HR (95% CI)	P
Age, year	1.02 (1.01-1.08)	0.030	0.98 (0.94-1.01)	0.208
Sex, male	1.10 (0.75-1.59)	0.619	–	–
SBP <90 mmHg	2.85 (1.08-7.51)	0.034	1.84 (0.56-5.95)	0.309
AKI at admission	2.52 (1.19-5.33)	0.015	1.11 (0.42-2.92)	0.835
NET > median	3.81 (1.43–10.16)	0.004	3.21 (1.08-9.19)	0.035
PWR	0.92 (0.88-0.96)	0.001	0.95 (0.89-1.00)	0.050
IL-6*	2.00 (1.35-2.95)	0.004	1.46 (0.93-2.30)	0.098
BNP > median	10.24 (3.10-33.95)	<0.001	5.92 (1.50-23.26)	0.011

*log transformed, AKI, acute kidney injury; PWR, platelet/WBC ratio; IL-6. Interleukin-6; BNP, B-type natriuretic peptide. "-" this variable is not included in the adjusted analysis.

### 3.4 Association between higher NET and AKI

Given the strong association between NET and AKI, the risk of COVID-19 AKI associated with higher levels of NET was investigated. In a logistic regression model, significant predictors of AKI in unadjusted analysis, were age >70 years (OR 2.58, 95% CI 1.17–5.65, p=0.018), pre-existing CKD (OR 4.21, 95% CI 1.82–9.76, p=0.001), higher IL-6 (OR 1.65, 95% CI 1.19–2.30, p=0.002), higher BNP levels (OR 2.31, 95% CI 1.05–5.04, p=0.036) and higher NET (OR 3.79, 95% CI 1.87–8.58, p=0.001). After model adjustment for age >70 years, sex, diabetes, pre-existing comorbidities, and IL-6 and BNP levels, a higher NET (OR 3.67, 95% CI 1.30–10.41, p=0.014), pre-existing CKD (OR 8.55, 95% CI 2.07–35.38, p=0.010) and increased IL-6 (OR 1.59, 95% CI 1.09-2.32, p=0.016) were significant determinants of COVID-19 AKI (model 3) (**Table 4**).

**Table 4:** Risk of AKI in association with high NET level.

	Unadjusted		Model 1		Model 2		Model 3	
AKI, at admission	OR (95% CI)	P	OR (95% CI)	P	OR (95% CI)	P	OR (95% CI)	P
NET > median	3.79 (1.87-8.58)	0.001	3.91 (1.70-9.01)	0.001	2.83(1.16-6.61)	0.023	3.67 (1.30-10.41)	0.014
Age >70 years	2.58 (1.17-5.65)	0.018	2.43 (1.05-5.66)	0.039	2.46 (1.02-5.95)	0.044	2.03 (0.62-4.54)	0.401
diabetes	1.13 (0.53-2.45)	0.435	1.22 (0.58-2.83)	0.632	1.10 (0.60-2.00)	0.580	0.90 (0.17-1.90)	0.555
Pre-existing CKD	4.21 (1.82-9.76)	0.001	–		3.84 (1.37-10.72)	0.010	8.55 (2.07-35.38)	0.010
IL-6	1.65 (1.19-2.30)	0.002	–		–		1.59 (1.09-2.32)	0.016
BNP > median	2.31 (1.05-5.04)	0.036	–		–		0.64 (0.40-1.02)	0.631

Model 1, adjusted with age>70, sex, and diabetes Model 2, model 1 + adjustment for pre-existing CKD, Model 3: model 4 + adjustment for BNP and ln_IL-6.

CKD, chronic kidney disease; IL-6. Interleukin-6; BNP, B-type natriuretic peptide. "-" this variable is not included in the adjusted analysis.

### 3.5 Comparison of nucleosome levels, neutrophil count and PWR for predicting prognosis

As the neutrophil count or the PWR at the time of admission was found to be as good as the nucleosome level in predicting clinical outcome, the sensitivity and specificity of these variables in predicting prognosis were compared using ROC analysis. We found that the AUCs of circulating nucleosome, peripheral neutrophil count, and P/W ratio were very similar; 0.678, 0.640, and 0.761 for mortality prediction and 0.681, 0.666, and 0.717 for AKI prediction, respectively. Pairwise comparisons showed that the difference between AUCs was not statistically significant, suggesting the usefulness of peripheral neutrophil count and PWR in predicting outcomes (**Supplementary Figure 1**).

## 4 Discussion

Our study provides evidence that elevated levels of NETs in patients with COVID-19 are closely associated with AKI, and that higher NET-related AKI is a strong predictor of higher mortality. We measured two markers of NETs, circulating nucleosomes and MPO-DNA, and found that both were significantly higher in patients with AKI than in those without AKI at admission. The urinary KIM/creatinine ratio and β2M were also significantly increased in patients with higher nucleosome levels than in those without, suggesting a role for NETs in renal injury. The increased risk of AKI with higher NET levels was independent of age, pre-existing CKD or inflammatory cytokine levels.

It was first proposed in 2004 that NETosis contributes to the first line of host defense against invading microorganisms (29). Although NETs have a protective role against pathogens, these complexes have been implicated in several thrombo-inflammatory conditions, including sepsis, thrombosis, and respiratory failure (1). Support for the pathogenic role of NETs comes from studies showing that the inhibition of neutrophils and NETs is protective in models of influenza-associated ARDS. This observation sparked interest in the role and clinical features of NETs in COVID-19 infections. The first detection of NETs in the plasma of SARS-CoV-2-infected patients was followed by reports of a significant role of NETs in COVID-19 (6). Elevated levels of blood neutrophils and NETs are an early indicator of SARS-CoV-2 infection, predicting severe respiratory disease and worse outcomes (6, 7, 30). In addition, neutrophils exposed to live SARS-CoV-2 develop NETs to a greater extent than other neutrophils, suggesting that SARS-CoV-2 infection is a pro-NETosis state (20). The virus itself as well as damaged epithelial cells, activated platelets, activated endothelial cells, and inflammatory cytokines are thought to trigger NETosis in COVID-19 (3, 8, 31).

As with the pulmonary complications of COVID-19, renal complications associated with COVID-19 have recently been reported. In particular, AKI is common, especially in ICU patients (32). In our patients, the prevalence of AKI at the time of admission was 36.5% and as high as 55% in patients admitted to the ICU (33). Consistent with other data (8, 18, 19), our study shows that COVID-19 AKI is associated with significantly higher rates of ICU admission, a greater need for MV, increased in-hospital mortality, and longer hospital stays. Recently, the 25th Consensus Conference of the Acute Disease Quality Initiative proposed that endothelial dysfunction, coagulopathy, tubular damage, and complement activation are key mechanisms of COVID-19 AKI (33–35). Intravascular NETs may also contribute to COVID-19 AKI by inducing microvascular inflammation and thrombosis (26). Indeed, endothelial dysfunction associated with microvascular damage and pro-thrombotic conditions leading to thrombotic occlusion of the renal microvasculature may be a key feature of NET-associated AKI (36, 37). In support of this, our data show that nucleosome levels correlate very strongly with a higher serum vWF levels, a marker of endothelial activation and damage. And the nucleosome and vWF levels were significantly higher in those with AKI than in those without.

We also found that patients with high nucleosome levels had significantly lower PWR than those with low nucleosome levels, and that low PWR predicted AKI and mortality as well as nucleosome levels. Like neutrophils, platelets also play an important role in the intravascular immune response. In COVID-19 patients, platelets coordinate with neutrophils to release NETs, which can induce platelet-neutrophil aggregates with thrombocytopenia (38, 39). These can activate the procoagulant cascade, which is associated with the prognosis of septic death and microvascular thrombosis and subsequent organ dysfunction (40, 41). Consistent with our findings, a recent study from Thailand found that PWR can be an accurate predictor of in-hospital mortality in patients with severe COVID-19 pneumonia (42). These findings suggest the importance and utility of neutrophil count and PWR in predicting clinical outcomes in COVID-19.

One of the hallmarks of COVID-19 infection is systemic inflammation leading to a cytokine storm (43, 44), which in turn has been implicated in severe multi-organ failure during acute viral infection (45, 46). High levels of pro-inflammatory cytokines are associated with more severe respiratory disease. Our study also shows significantly higher serum levels of IL-6, MCP-1, and procalcitonin in COVID-19 patients who died than in those who survived, but surprisingly, plasma nucleosome levels were not associated with IL-6 and MCP-1 levels. In contrast to our findings, Zuo et al. reported that cell-free DNA is strongly associated with CRP levels in patients with severe COVID-19 infection (6, 26). The difference in results may be due to the fact that cell-free DNA is a less specific marker of NETs and therefore may correlate with the overall level of inflammation, rather than being specific to neutrophil activation. Another possible explanation for the difference is that serum IL-6 or MCP-1 levels may not fully represent the overall immune activation status in COVID-19 infections. In fact, circulating levels of IL-6 are significantly lower in patients with COVID-19 than in patients with sepsis (47). This suggests that cytokines are only moderately elevated in COVID-19 and are therefore unlikely to have a significant relationship with NET levels in these patients (33). Another alternative explanation is that NETs are more closely associated with endothelial damage and coagulation dysfunction rather than representing a cytokine storm, although the latter may be involved in significant crosstalk with NETs (21). It is also possible that the close association between NETs and the hyperinflammatory response is only seen in severe cases. Further research is needed to fully understand their interactions, as well as the role of systemic inflammation and NET formation in COVID-19 AKI.

This study had several limitations. First, blood samples were taken at the time of admission, but the duration of the disease from the onset of symptoms will have varied greatly given the differences in the severity of symptoms between patients. In addition, some patients were transferred from another hospital during their treatment. Second, it was not possible to adjust for the effect of antiviral therapy in predicting prognosis because the dose and duration of antiviral agents used depended on the patient’s clinical situation. Third, NETs may have been partially degraded over time, which would have affected their measurement. Fourth, it would be helpful to compare circulating nucleosome levels between non-hospitalized and hospitalized COVID-19 patients to show the prognostic significance of high nucleosome levels. However, it was practically difficult to collect blood and urine samples from non-hospitalized COVID-19 patients, most of whom are in quarantine. Fifth, the diagnostic or prognostic cut-off value of circulating nucleosomes has not yet been established. Therefore, we arbitrarily divided the patients into 2 groups according to the median value. Finally, a causal relationship between COVID-19 AKI and NETs could not be established in this study, nor could we evaluate the long-term effects of NETs or AKI on mortality after patient discharge. Furthermore, the relationship between NETs and COVID-19 vaccination and the prognostic role of NETs in COVID-19 patients with different vaccination histories is uncertain. Future studies should investigate the predictive power of circulating NETs and NET-related AKI in well-characterized longitudinal cohorts with different vaccination histories.

In conclusion, elevated levels of NETs in patients with COVID-19 can predict in-hospital mortality. In addition, higher NETs were significant determinants of COVID-19 AKI, independent of age, pre-existing CKD, and inflammatory cytokine levels. Circulating nucleosome levels were strongly associated with higher vWF, but not with inflammatory cytokine levels, suggesting a role for NETs in endothelial injury or coagulopathy in the development of COVID-19 AKI. Our results suggest a role for NET-related AKI in NET-related mortality, mediated by vascular injury and inflammation. However, a causal relationship between NETs and AKI or poor outcomes remains to be established.

## Data availability statement

The raw data supporting the conclusions of this article will be made available by the authors, without undue reservation.

## Ethics statement

The studies involving human participants were reviewed and approved by HALLYM 2022-02-014-001. The patients/participants provided their written informed consent to participate in this study.

## Author contributions

J-KK and YK: Study design, set-up, data analysis and interpretation. J-KK, IK and HL: acquisition of experimental data and writing-up. DK: patient recruitment and clinical data acquisition. SK: Data analysis and statistical advisory. All authors contributed to the article and approved the submitted version. 
