# Supplementary figures and images for: Role of increased neutrophil extracellular trap formation on acute kidney injury in COVID-19 patients
Source: Front Immunol. 2023 Mar 27;14:1122510. doi: 10.3389/fimmu.2023.1122510 (PMC10083414; doi:10.3389/fimmu.2023.1122510)

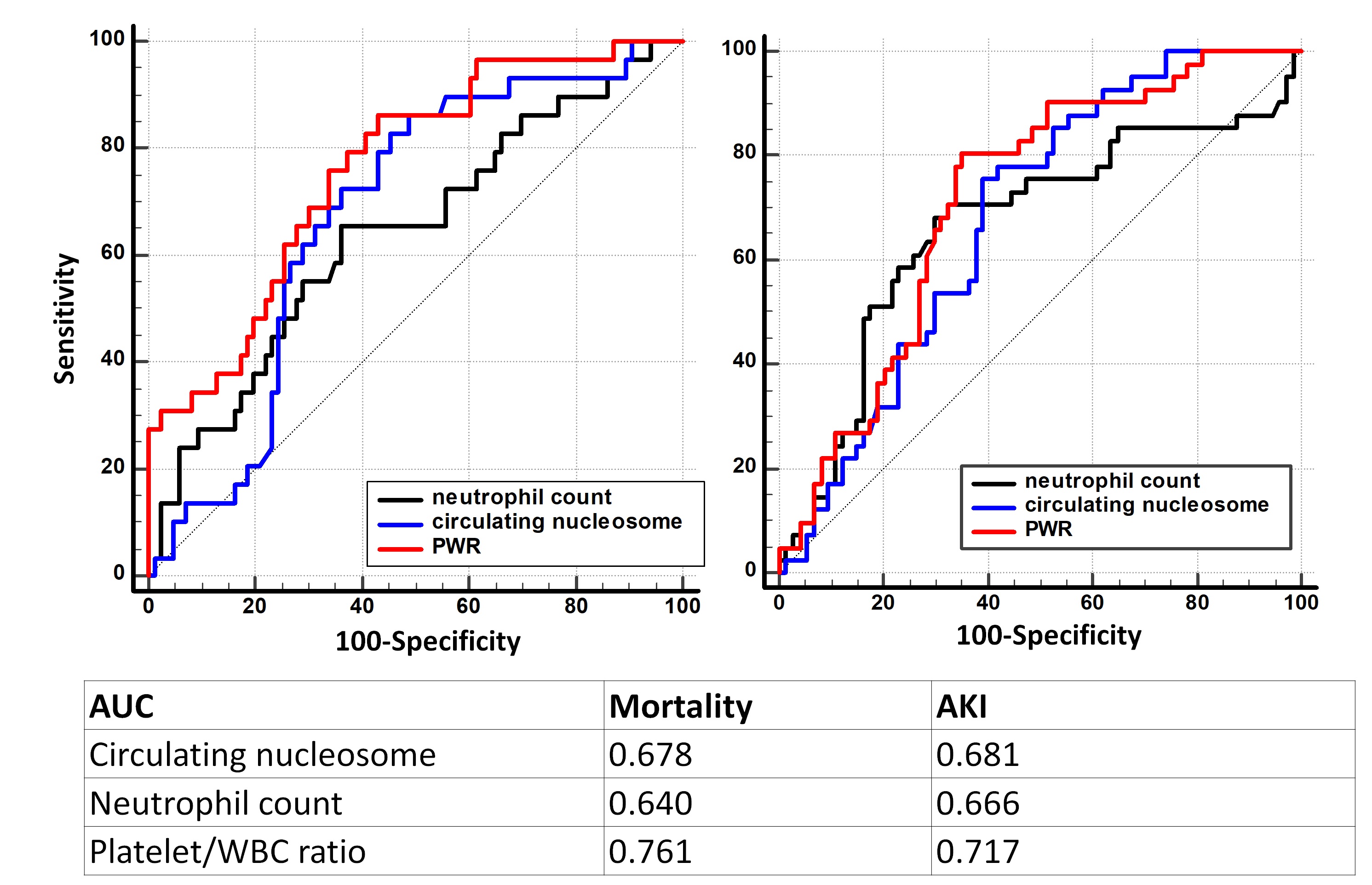

Supplement: Supplementary file 1 [file Image_1.jpeg]
